# Exploring the role of AI in dental education: a mixed-method experimental study from Pakistan

**DOI:** 10.1186/s12909-026-08728-4

**Published:** 2026-02-10

**Authors:** Tahera Ayub, Muhammad Shahid Shamim, Rahila Ali, Syed Shirjeel Husain

**Affiliations:** 1 Department of Medical Education, Liaquat College of Medicine and Dentistry, Karachi, Pakistan; 2 Department for Educational Development, Aga Khan University, Karachi, Pakistan; 3 Department Medicine, Liaquat College of Medicine and Dentistry, Darul Sehat Hospital, Karachi, Pakistan

**Keywords:** Artificial intelligence, Case-based learning, Dental education, Mixed-methods study, ChatGPT, Health professions education, Pakistan, Educational technology, Clinical reasoning, Student perception

## Abstract

**Background:**

Case-based learning (CBL) is a cornerstone of dental education, fostering critical thinking and clinical reasoning. In Pakistan, challenges like low facilitator-to-student ratios and faculty shortages hinder CBL effectiveness. Artificial intelligence (AI) offers potential solutions through scalable, structured facilitation, yet its role in dental CBL remains underexplored.

**Objective:**

The objective of this study was to evaluate the effectiveness and acceptability of artificial intelligence (AI) as a facilitator in case-based learning (CBL) for dental education. It aimed to compare learning outcomes between AI- and human-facilitated sessions using a randomized crossover design, and to explore students’ perceptions and experiences of AI-mediated learning through focus group discussions.

**Methodology:**

A sequential mixed-methods design (QUAN → QUAL) was employed with 16 final-year BDS students in a randomized two-period crossover trial. Phase 1 compared AI-facilitated (via ChatGPT-3.5) and human-facilitated CBL using pre- and post-test MCQs, analyzed with Wilcoxon signed-rank and Mann-Whitney U tests. Phase 2 explored student experiences through focus group discussions (FGDs), analyzed thematically using Braun and Clarke’s framework. Ethical approvals were obtained (ERC #2023-6789-12345, IRB-789/LCMD/2023).

**Results:**

Both AI- and human-facilitated groups showed significant post-test score improvements (*p* < 0.012), with no significant inter-group differences (*p* ≥ 0.130). AI excelled in factual recall and structured feedback, while human facilitation enhanced emotional engagement and clinical reasoning. FGDs revealed themes of AI’s consistency but limited emotional depth, with students favoring a hybrid model.

**Conclusion:**

AI-facilitated CBL yields comparable academic outcomes to human facilitation, offering scalability in resource-limited settings. However, human facilitators remain vital for emotional and adaptive learning. A blended approach, integrating AI’s structure with human mentorship, is recommended for optimal dental education.

## Introduction

Case-based learning (CBL) is widely recognized as an effective teaching and learning tool in medical education [1]. It prepares students for clinical practice’s uncertainty and variability, developing essential adaptability, critical thinking and problem-solving skills for successful practice. Facilitated by a trained faculty, it can effectively engage students in discussing real-world clinical scenarios, even within limited teaching spaces, to deepen theoretical knowledge and reasoning [2].

In Pakistan, however, implementation of CBL in dental education faces several challenges, primarily due to structural and systemic constraints. Key among these include low facilitator-to-student ratios, variability in facilitation quality and faculty time constraints, leading to inconsistent learning outcomes [3, 4]. The rapid proliferation of dental institutions across the country, without a corresponding increase in qualified faculty, has further exacerbated these issues. These factors have overstretched the resources and reduced the quality and effectiveness of CBL. The issues may be resolved by recent developments in Artificial Intelligence (AI) supported systems, which offer reliable, scalable, and flexible instructional help.

AI has been increasingly employed in several ways to enhance educational delivery in medical education by offering potential solutions to these challenges. AI-powered tools, such as intelligent tutoring systems and virtual patients, have been successfully used to deliver consistent practice scenarios and educational support in various domains, reducing the need for human oversight [5]. These technologies can to present clinical situations for personalized learning and tailored feedback, significantly enhancing the CBL experience [6]. However, their integration into dental education, particularly as a supplement or alternative to human facilitation in CBL, remains underexplored.

AI-based facilitators can ensure more equitable access to structured, interactive, and contextually relevant learning experiences [9]. Technological advances in AI-supported learning systems may address key limitations in current CBL implementation in dental education. By mimicking elements of human facilitation, such as assisting in decision-making, supporting reflection, AI tools can help maintain the pedagogical integrity of CBL while mitigating faculty shortages and inconsistencies These tools can mimic human facilitation [7].

This study, therefore, explores the use of AI as a supportive resource to fill the gap by strengthening the delivery of CBL in dental education, particularly in contexts where access to qualified facilitators is limited. The objective of this study was to evaluate the effectiveness and acceptability of artificial intelligence (AI) as a facilitator in case-based learning (CBL) for dental education. Specifically, it sought to compare learning outcomes between AI-facilitated and human-facilitated sessions using a randomized crossover design, while also exploring students’ perceptions and experiences of AI-mediated learning through focus group discussions.

Based on this objective, the null hypothesis stated that there is no significant difference in learning outcomes between AI-facilitated and human-facilitated CBL sessions, and that student’s perceptions of AI-mediated facilitation do not differ meaningfully from those of human facilitation.

## Methods

### Study design

The study complies with the Helsinki Declaration under the *Ethics approval and consent*. Ethical clearance was obtained through the Institutional Review Board (IRB) approval letter (Ref: IRB/D-000077/23), along with a No Objection Certificate (NOC) for data collection from students was obtained from the institution (Ref: LCMD/BDS/PO/LET-24/00271), and Aga Khan University Ethical Review Committee (ERC #2023-6789-12345). Written informed consent was obtained individually from each participant prior to their inclusion in the study. The study was conducted at Liaquat College of Medicine and Dentistry (LCMD) in 2024, Karachi, involving final-year BDS students during their clinical rotation in the Oral and Maxillofacial Surgery department. Willing to participate in both the Case-Based Learning (CBL) sessions and the subsequent Focus Group Discussions (FGDs). Students who had prior exposure to AI-facilitated CBL sessions (to avoid bias from previous experience with the intervention) were excluded.

This study employed a sequential mixed-methods design (QUAN → QUAL) to evaluate the use of artificial intelligence (AI) as a facilitator in case-based learning (CBL) in dental education. Conducted in two phases, the study first measured and compared learning outcomes between AI-facilitated and human-facilitated CBL sessions (Phase 1), followed by an in-depth exploration of students’ perceptions and experiences through focus group discussions (Phase 2).

#### Phase 1: quantitative experimental study

A randomized two-period crossover design was used to objectively compare learning outcomes from AI-facilitated and human-facilitated CBL. A total of 16 final-year Bachelor of Dental Surgery (BDS) students were randomly allocated into two groups (*n* = 8 each) using computer-generated randomization. The study involved sixteen final-year BDS students during their clinical rotation in the Oral and Maxillofacial Surgery department. Participants were recruited through convenience sampling. The students were randomly divided into two groups of eight each and allocated to AI-mediated or human-facilitated sessions using a two-period crossover design with a one-week washout period to enhance validity.

**Fig. 1:**
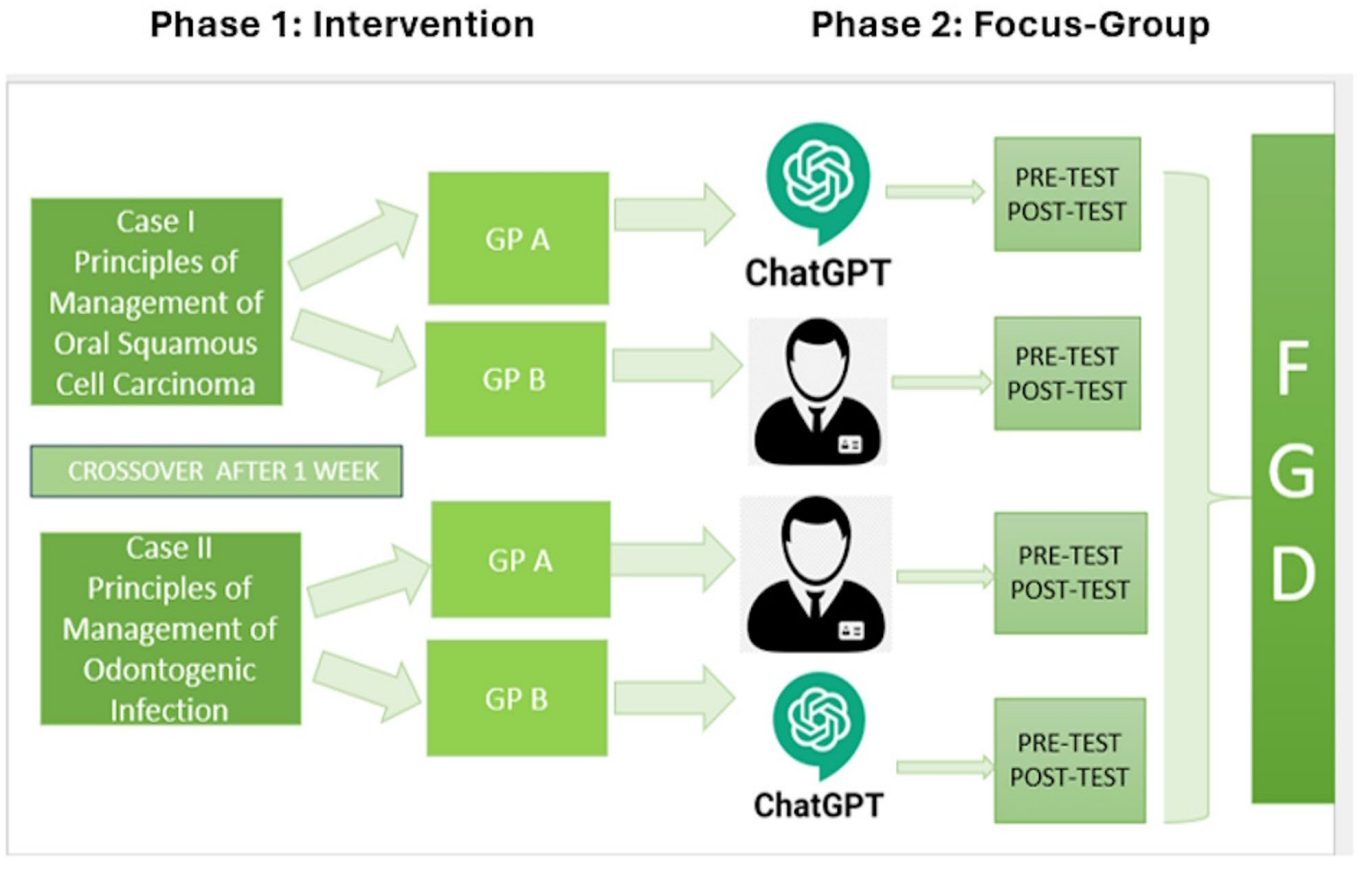
Study overview illustrating the two sequential phases: Phase 1 (Intervention) and Phase 2 (focus-group)

Two clinical cases were employed, vetted for validity by an expert panel with over five years of teaching experience. The same cases were used across both modalities to ensure comparability. Group A began with AI-facilitated CBL delivered through a structured ChatGPT-3.5 interface programmed to replicate guided facilitation, then crossed over to human facilitation. Group B followed the reverse sequence, starting with human facilitation before crossing to AI (Fig. 1).

Sessions were conducted under observation to ensure protocol fidelity. The same cases and assessment tools were used across both modalities to control for content variation. Pre- and post-tests using validated multiple-choice questions (MCQs) assessed learning outcomes.

##### Sample size

A power analysis using G*Power (version 3.1) estimated the minimum sample size required for a paired comparison of pre- and post-test scores. Assuming a medium effect size (Cohen’s d = 0.5), α = 0.05, and power = 0.80, a sample of 16 participants was deemed sufficient. This aligns with prior crossover studies in educational interventions.

#### Phase 2: qualitative exploratory study

Following the quantitative phase, focus group discussions (FGDs) were conducted to explore student perceptions of AI versus human-facilitated CBL. Two FGDs (*n* = 8 participants each) were held in a neutral setting and moderated by an experienced facilitator using a semi-structured guide. Discussions were audio-recorded, transcribed verbatim, and subjected to thematic analysis following Braun and Clarke’s six-step approach [8].

### Data collection and analysis

#### Quantitative analysis

Descriptive statistics (mean, SD, median, and range) were calculated for pre- and post-test scores. The Shapiro-Wilk test assessed data normality. Within-group comparisons of learning gains were analyzed using the Wilcoxon signed-rank test, while between-group comparisons of post-test scores employed the Mann-Whitney U test. MCQ reliability was assessed using Fleiss’ Kappa across three expert raters, showing near-perfect agreement for Case 1 (κ = 0.83, *p* < 0.001) and moderate agreement for Case 2 (κ = 0.76, *p* < 0.001).

#### Qualitative analysis

FGD transcripts were reviewed independently by two coders. Member checking was used to validate transcript accuracy with participants. Thematic analysis was conducted inductively, following the Braun and Clarke framework: familiarization, initial coding, theme development, and interpretation. Field notes and reflective memos supplemented the data, enhancing analytical rigor. Inter-rater reliability was high (Cohen’s κ = 0.81), and intra-rater reliability was also strong (κ = 0.89).

Six major themes emerged:

1. Engagement and Interaction Dynamics: Capturing the initial excitement, student engagement, and interactive nature of AI-mediated versus human-facilitated CBL, while noting the limitations in personal touch and responsiveness.
2. Structured Learning and Depth of Understanding: Highlighting AI’s systematic approach, its contribution to factual recall and structured feedback, contrasted with human facilitation’s strength in clinical reasoning and adaptive questioning.
3. Technical Challenges and Adaptation: Addressing issues related to interface usability, technical glitches, and the process of student adaptation to AI-led facilitation, alongside preferences for hybrid models.
4. Emotional Resonance and Personal Connection: Exploring the motivational impact of human interaction, the lack of emotional depth in AI facilitation, and the importance of personal connection in learning experiences.
5. Future Potential and Evolution of AI in Education: Reflecting on students’ recognition of AI’s growing role, its potential for enhancement, and the need for continuous evolution to meet educational demands.
6. Personal Growth and Changing Perspectives: Emphasizing how exposure to both facilitation methods influenced students’ self-reflection, broadened perspectives, and contributed.

Triangulation of data sources (MCQs, FGDs, field notes) enhanced validity, and integration of qualitative findings contextualized quantitative results by explaining the observed learning patterns and student attitudes toward each modality.

## Results

### Quantitative analysis

A total of 32 observations were collected from 16 study participants across two CBL sessions. The mean age of participants was 22.19 years (SD ± 0.83), with a male-to-female ratio of 5:3.

### Intra-group comparison of CBL sessions

Participants in both the AI-facilitated and human-facilitated groups demonstrated statistically significant improvements in post-test scores across both CBL sessions. In Session 1, Group A (AI-facilitated) showed a median improvement from 20 (IQR 3.5) to 27 (IQR 2.75) (*p* = 0.011), while Group B (human-facilitated) improved from 18 (IQR 5.25) to 27.5 (IQR 3.5) (*p* = 0.012). Following the crossover, Group A (now human-facilitated) improved from 16 (IQR 3.5) to 26.5 (IQR 4.75) (*p* = 0.011), and Group B (now AI-facilitated) from 24 (IQR 3.25) to 28.5 (IQR 1.75) (*p* = 0.010) (Table 1). The IQR report the variability in students performance. In Session 1, Group A (AI-facilitated) demonstrated a reduction in IQR from 3.5 to 2.75, indicating that post-test scores were more tightly clustered around the median, reflecting greater consistency in learning outcomes. Group B (human-facilitated) also showed a reduction in IQR from 5.25 to 3.5, suggesting decreased variability and improved uniformity of performance after facilitation. In Session 2, Group A (now human-facilitated) exhibited an increase in IQR from 3.5 to 4.75, implying wider variability in post-test scores and less consistency among participants. Conversely, Group B (now AI-facilitated) showed a marked reduction in IQR from 3.25 to 1.75, highlighting highly consistent performance across students following AI facilitation.

**Table 1:** Intra-group comparison of pre- and post-test scores within groups (*N* = 16)

Session	Group	Intervention	Pre-Test Median (IQR)	Post-Test Median (IQR)	*p*-value
CBL 1	A (*n* = 8)	AI	20 (3.5)	27 (2.75)	0.011*
	B (*n* = 8)	Human	18 (5.25)	27.5 (3.5)	0.012*
CBL 2	A (*n* = 8)	Human	16 (3.5)	26.5 (4.75)	0.011*
	B (*n* = 8)	AI	24 (3.25)	28.5 (1.75)	0.010*

*Wilcoxon Signed-Ranks Test

### Inter-group comparison

In Session 1, where Group A was AI-facilitated and Group B was human-facilitated, there was no significant difference in pre-test scores (*p* = 0.050) or post-test scores (*p* = 1.000) between groups. In Session 2, Group B (AI-facilitated) had significantly higher pre-test scores than Group A (human-facilitated) (*p* < 0.001), pretest score both groups had similar central tendency (medians close), but Group B showed greater variability (wider IQR). The *p*-value (0.050) suggests no statistically significant difference, though Group A’s scores were slightly more consistent. but post-test scores medians are nearly identical, and variability is low in both groups. The *p*-value (1.000) confirms no difference, showing both facilitation methods led to comparable and stable learning outcomes (Table 2).

**Table 2:** Inter-group comparison of pre- and post-test scores between groups (*N* = 16)

Session	Group	Pre-Test Median (IQR)	*p*-value	Post-Test Median (IQR)	*p*-value
CBL 1	A (AI)	20 (3.5)	0.050*	27 (2.75)	1.000*
	B (Human)	18 (5.25)		27.5 (3.5)	
CBL 2	A (Human)	16 (3.5)	< 0.001*	26.5 (4.75)	0.130*
	B (AI)	24 (3.25)		28.5 (1.75)	

*Mann-Whitney U Test

### Performance by item difficulty

Analysis by item difficulty level showed improvement in correct responses across all categories for both facilitation types. The AI-facilitated group showed increases from pre- to post-test as follows: easy questions from 37.5% to 44.4%, moderate from 36.9% to 45.3%, and difficult from 32.4% to 43.2%. Similarly, the human-facilitated group improved from 29.7% to 41.7% (easy), 29.4% to 44.1% (moderate), and 25.8% to 42.3% (difficult). The AI group outperformed the human group slightly across all difficulty levels.

### Performance by cognitive level

When categorized by cognitive complexity, AI-facilitated learners showed gains from 52.5% to 66.0% (C2) and from 54.3% to 66.9% (C3). The human-facilitated group also improved from 40.2% to 65.4% (C2) and from 44.7% to 62.7% (C3). This indicates that both facilitation methods effectively enhanced higher-order cognitive skills.

### Qualitative analysis

Six major themes with corresponding subthemes were derived from focus group discussions, reflecting students’ experiences with both AI and human facilitation. In this Table 3, codes represent specific ideas or expressions derived from participant’s statements. These codes were clustered into subthemes, which in turn were synthesized into broader themes. This hierarchical structure (Codes → Subthemes → Themes) illustrates the inductive process of thematic analysis.

**Table 3:** Thematic outcomes from focus group discussions

Theme	Subthemes	Codes
Colossal Leap of the New Millennium	Initial Excitement, Engagement & Interaction, Lacking Personal Touch	Novelty of AI, Technological intrigue, Rapport building, Dynamicity, Revising alone, Impersonal interaction, Understanding cues
Journey to Unearth Mountains	AI’s Structured Approach, Depth of Learning, Feedback	Structured format, Consistent content delivery, Prompt feedback, Superficial engagement, Critical thinking
Rome Was Not Built in a Day!	Technical Issues, Adaptation to AI	Technical glitches, Learning curve, User interface
Humans! The Master Creations	Lack of Emotional Depth, Personal Connection in Learning	Missing empathy, Emotional support, Human facilitator’s influence
Another Edmund’s Challenge: Everest of AI	Future Potential of AI, Need for Evolution	AI in diagnostics and education, Improved responsiveness, Understanding nuance
Evolution as a Learner and Thinker	Enhancement of Personal Growth, Change in Perspectives	Self-directed learning, Reflective practices, Boosted confidence, Improved analytic skills

### Theme 1: colossal leap of the new millennium

Students expressed initial excitement and curiosity about AI-based learning. One remarked, *“When we started with the AI*,* it was fascinating. The way it presented cases and interacted was unlike anything we’d done before”*. Another noted, *“There was this one session with the AI*,* and I was surprised. It asked us*,* ‘Would you like to explore more on this topic or review what we’ve covered?”*.

Despite enthusiasm, some students identified limitations in AI facilitation. *“Every session was well-structured*,* but it felt somewhat mechanical*,* lacking the nuances of human interaction”*, and *“That’s true*,* but there were times I wished the AI could sense our struggle a bit more”*. Students compared this with human facilitation: *“Our human facilitator could immediately sense when we were lost and would adjust the discussion*,* making it more than just a learning experience but a shared journey”*.

### Theme 2: journey to unearth mountains

Students highlighted the AI’s structured approach and its impact on their thinking. One commented, *“The AI suggested a completely different approach to the problem. It wasn’t what I expected*,* but it opened up a new way of thinking for me”*. Another noted, *“Every session was well-structured”*, appreciating its consistency.

However, some felt the methodical nature limited depth: *“It was all very methodical*,* but I sometimes felt like we were just going through the motions without really delving deep into the problems”*. Yet students recognized academic benefits: *“The AI’s structured approach to dissecting cases has refined my analytical skills”*.

### Theme 3: Rome was not built in a day!

Technical challenges and a learning curve were common. One student shared, *“There were a few hiccups like the AI not responding or misunderstanding our queries”*, suggesting prior input to improve interactions. Another noted, *“Improving AI’s user interface to make it more engaging and less intimidating can also help. It was frustrating at times*,* especially when we were trying to keep up with the session”*.

Students gradually adapted, learning how to interact effectively: *“It took a couple of sessions to get the hang of it*,* to learn how to phrase our questions effectively”*.

### Theme 4: Human! The master creations

Students consistently emphasized the value of human interaction and empathy. *“You don’t realize how much you value a teacher’s empathy until it’s replaced by AI”* and *“AI can teach*,* but it missed cues that a human teacher would notice”*. Personal connection was important: *“With our human facilitator*,* I felt more connected and involved”* and *“Our facilitator brought real-life experiences that made learning richer”*.

### Theme 5: another edmund’s challenge; everest of artificial intelligence

Students acknowledged AI’s potential while noting its limitations. *“AI can be useful in patient education and diagnosis*,* but it still can’t replace a dentist’s clinical judgment”*. The use of simulations was highlighted: *“It would be great if AI could simulate patient interactions to apply what we’ve learned”*. Limitations included cultural sensitivity and social understanding: *“AI still lacks the ability to fully understand the emotional and social dynamics of real patients”*.

### Theme 6: evolution as a learner & thinker

AI gradually became a tool that enhanced learning and analytical skills. *“I was hesitant at first*,* but AI provided a judgment-free zone where I could ask questions without fear”*, and *“AI gave me time to think and reflect; it didn’t rush me like a regular class might”*. Students acknowledged AI as a supplement: *“Our teachers understand our emotions; AI can’t do that*,* but it helped me sharpen my reasoning”*. The findings support a blended model where AI complements human facilitation to optimize learning.

## Discussion

The quantitative findings of this sequential mixed-methods study demonstrate that both AI-facilitated and human-facilitated case-based learning (CBL) sessions resulted in statistically significant improvements in students’ post-test scores (*p* ≤ 0.012 across all intra-group comparisons), with no significant differences in post-test performance between the two modalities (*p* ≥ 0.130). These results support the null hypothesis that there is no significant difference in learning outcomes between AI- and human-facilitated CBL, confirming that AI, using ChatGPT-3.5 as a structured facilitator, can achieve comparable knowledge gains to traditional human facilitation in dental education. The comparable performance between human and AI facilitation suggests that AI holds potential as an effective educational tool in dental education, particularly in resource-limited settings. The finding is in line with the MaMa et al., who described an AI-driven design that significantly improved various aspects of intercultural competence in a mixed-methods study using pre- and post-test measures, revealing significant enhancements in student’s cultural knowledge, communication skills, and overall engagement [9]. Contrary to this, CBL mostly depends on contextual judgment, clinical reasoning, and dynamic human interaction, skills that AI cannot completely replace. The integration of AI applications in dentistry into complex instructional formats like CBL is hampered by issues including low data generalizability, a lack of methodological rigor, and ethical considerations [10]. Additionally, the FDI World Dental Federation stresses that although AI can assist education by simulating and analyzing data, it cannot take the place of the critical thinking and cooperative discussion that are crucial to CBL [11].

Students responded positively to AI’s structured approach, appreciating its systematic guidance. One student shared, *“When we started with the AI*,* it was fascinating. The way it presented cases and interacted was unlike anything we’d done before”*. These reflections highlight the novelty, curiosity, and critical thinking prompted by AI facilitation. This is similar with research by Liu et al. [12]and Mian and Khan [13], who emphasized AI’s capacity to provide constant feedback and round-the-clock accessibility, promoting self-paced learning and greater student autonomy.

The structured nature of AI-led sessions was seen as both an advantage and limitation. Nonetheless, students appreciated the alignment of AI-generated content with their academic curriculum. This is in line with M.Yang et al. [14], who found that because AI-facilitated learning environments were structured, they decreased cognitive overload and increased task completion rates. Contrarily, AI systems often lack the emotional intelligence that human educators possess. According to Kim et al., teachers have expressed concern about the potential for AI to replace human interactions within educational frameworks, which they perceive as a loss of the human elements essential for effective teaching and learning [15].

### Comparable effectiveness across facilitation modes

Both AI- and human-facilitated sessions led to statistically significant improvements in post-test scores, consistent with CBL’s established effectiveness in dental education. These results align with prior studies indicating that AI-supported instruction can achieve learning outcomes comparable to traditional pedagogy. Muralidharan et al. demonstrated that technology-aided instruction designed to support academically weaker students resulted in substantial gains in test scores, underscoring the potential of AI in customizing learning experiences to meet individual needs [16]. AI facilitated learning across different cognitive domains, supporting factual recall and analytical reasoning.

However, AI was sometimes perceived as impersonal and mechanically structured. While AI efficiently delivered content, its capacity to adapt explanations or address conceptual ambiguity was limited. This sentiment is echoed by Herawati et al., who noted that while many students view AI as a tool that can enrich their learning experiences, there are substantial concerns regarding the mechanization of educational processes and the diminishing role of teachers [17]. The emotional engagement that comes from personalized interactions with educators cannot easily be replicated by AI, leading to perceptions of AI as impersonal.

### Divergent learner experiences

Despite similar test score gains, students reported qualitative differences between AI- and human-facilitated sessions. AI’s novelty, speed, and structured feedback were valued. Prompt feedback was particularly appreciated. At the same time, students highlighted the limitations of AI, particularly its lack of emotional engagement and ability to perceive non-verbal cues. Cultural sensitivity and understanding social dynamics were also cited as areas where AI fell short: *“AI still lacks the ability to fully understand the emotional and social dynamics of real patients”*. These observations underscore the irreplaceable role of human facilitators in promoting trust, engagement, and reflective dialogue. AI interaction’s lack of emotional nuance and empathy, as it lacks empathy and warmth. This bolsters the criticism made by Shahroom et al. [18] Zarei et al. [19], who contended that AI is devoid of the socio-emotional intelligence necessary to establish rapport and provide a psychologically secure learning environment. In medical and dental education, where professional communication, empathy, and ethical reasoning are fundamental skills, these emotional indicators are crucial.

Overall, students anticipate a growing role for AI in dental education, but stressed the need for its evolution, highlighting the importance of making AI more adaptive, context-aware, and emotionally intelligent. These findings echo prior research underscoring the irreplaceable role of social and emotional dynamics in learning environments, especially in collaborative formats like CBL [20]. While AI can enhance delivery and feedback, it struggles to replicate the empathetic, responsive qualities that human facilitators bring to teaching.

### Structural clarity vs. depth of engagement

Students consistently noted AI’s structured and predictable approach as helpful in maintaining discussion flow. Yet, the same predictability sometimes limited critical engagement. Human-led sessions were more flexible and exploratory:

Feedback from AI was timely, analytical, and occasionally more specific than expected. This demonstrates AI’s potential to support individualized learning while highlighting a trade-off between consistency and depth of engagement. Similarly, Altınay et al. raised concerns regarding the restrictions AI could impose on teaching professions. They noted that while AI can support learning by addressing students’ needs, it may also discourage innovative teaching practices due to its often rigid framework [21].

### Practical and pedagogical implications

AI-facilitated CBL may serve as a viable supplement or alternative to human-led sessions, especially for foundational content and in resource-constrained environments. Technical challenges and the initial learning curve were noted by students: *“There were a few hiccups like the AI not responding or misunderstanding our queries”*, Gradual adaptation improved usability, with students suggesting enhancements for more nuanced responses in complex discussions.

Despite AI’s efficiency, the human touch remains critical for clinical excellence. Empathy, emotional support, and personalized guidance cannot currently be replicated by AI. Chen et al. underscore that AI technologies can tailor learning content to meet certain academic needs, but often lack the interpersonal aspects of mentorship that foster critical thinking, motivation, and self-confidence [22]. Such personal engagement is key to guiding students through their learning journeys in a way that feels supportive and individualized.

This notion is supported by Castro et al., who address the potential for AI-driven learner profiling and adaptive pathways [23]. They note how AI can recognize and adjust to learner preferences based on interaction data; however, it cannot cultivate the deeper, emotionally supportive relationships that characterize effective learning environments.A hybrid model leveraging AI for structure and efficiency, while retaining human facilitators for mentorship and deeper inquiry, appears to be the most pedagogically effective approach in dental education.

These findings provide compelling evidence in favor of a blended or hybrid CBL approach, in which educators step in to address more complex ethical, emotional, or clinical reasoning while AI tools support human facilitators by handling routine or factual questions and organizing discussions. This is consistent with the opinions of Popenici and Kerr [24], who support “intelligent augmentation” as opposed to AI replacing teachers. Additionally, a study on AI in gamified blended learning environments highlights the efficacy of AI-driven personalized learning paths in enhancing language acquisition and literacy skills among students in Shanxi, China [25]. The integration of gamification further motivates students by making learning more engaging and less daunting.

However, detractors like Selwyn [26] warn against relying too much on AI in higher education, claiming that it could diminish opportunities for student-faculty mentorship, which is crucial for professional development in the health sciences, and support a mechanistic view of learning.

Our study adds to the expanding conversation on integrating AI in education. It cautions about AI’s limitations in promoting comprehensive, human-centered learning while reaffirming its value in standardizing and expanding educational experiences. The analysis shows that although students are receptive to incorporating AI into their education, they are also aware of its limitations at present. The results affirm a blended model of education in which AI is used as an adjunct to, not a substitute for human facilitators, optimizing both technological efficiency and human empathy in clinical education. To ensure that both technological efficiency and human wisdom shape the future of dentistry education, CBL may well be best served by strategically merging AI with sympathetic, skilled human facilitators.

### Limitations and future directions

This study involved a small cohort from a single institution, limiting generalizability. The short-term nature of the intervention and the use of MCQs also constrain conclusions about long-term learning, clinical reasoning, or affective development. Even though the AI tool was a working prototype, it might not accurately reflect the range and potential of current AI learning platforms. Furthermore, despite scientific rigor, interpretation bias is a risk associated with the subjective nature of qualitative data analysis. It’s also possible that during group talks, some individuals repressed critical opinions out of social desirability or peer presence. Larger, multi-center studies with longitudinal follow-up that address these limitations in future research may offer more thorough insights into how AI is changing dental education.

## Conclusion

The results of the study showed that both AI-facilitated and human-facilitated CBL sessions led to a significant improvement in student’s post-test performance. AI-facilitated CBL sessions resulted in comparable academic outcomes to those led by human instructors. However, students perceived clear differences in emotional engagement, depth of interaction, and learning dynamics. While AI offers a promising tool for structured and scalable education, it cannot fully replicate, at least at this point in time, the human elements that foster trust, empathy, and adaptive learning. Educators and institutions should thus consider AI as a complementary, not replacement, tool in health professions education.

## Data Availability

All data generated or analyzed during this study are included in this published article.The datasets used and/or analyzed during the current study are available from the corresponding author on reasonable request.
